# Atrial proarrhythmic effect of lead as one of the PM_10_ metal components of air pollution. An in-silico study

**DOI:** 10.1371/journal.pone.0258313

**Published:** 2021-10-12

**Authors:** Laura C. Palacio, Diana C. Pachajoa, Geraldine Durango-Giraldo, Camilo Zapata-Hernandez, Juan P. Ugarte, Javier Saiz, Robison Buitrago-Sierra, Catalina Tobón

**Affiliations:** 1 MATBIOM, Universidad de Medellín, Medellín, Colombia; 2 GI^2^B, Instituto Tecnológico Metropolitano, Medellín, Colombia; 3 MATYER, Instituto Tecnológico Metropolitano, Medellín, Colombia; 4 GIMSC, Universidad de San Buenaventura, Medellín, Colombia; 5 CI^2^B, Universitat Politècnica de València, Valencia, Spain; University of Essex, UNITED KINGDOM

## Abstract

Particulate matter (PM) is considered the most severe environmental pollution problem due to its serious effects on human health associated with an increased risk of cardiovascular morbidity and mortality. In this work, a physicochemical characterization of PM_10_ from the city of Medellin was developed. The results evince that lead (Pb) is one of the most abundant elements since it is present in all analyzed samples. Therefore, Pb was chosen to perform an in-silico study to assess its effects on atrial arrhythmias generation. For this purpose, we developed a model representing the Pb^2+^ blocking effect on the L-type calcium channel. This formulation was incorporated in a human atrial cell mathematical model and in 2D and 3D models of human atria. The simulations showed a proarrhythmic effect at high Pb^2+^ concentrations, through shortening of action potential duration inducing the generation of reentrant activity and atrial flutter. The results contribute to the knowledge about the cardiac physiopathological processes, triggered by lead as one of the main PM_10_ metal components of air pollution, that yields the generation of arrhythmias.

## Introduction

Air pollution is responsible for around 4.3 million premature deaths each year [1]. The Global Burden of Diseases Study 2015, identified that air pollution is one of the main causes of morbidity worldwide, especially in low and middle income countries [2]. The main susceptible population to develop health problems as a result of air pollution are older people, children, and people with heart and lung diseases [1]. There is evidence that the several years reduction of life expectancy and an increased hospital admission due to cardiovascular diseases, are associated with prolonged exposures to air pollution [3]. Furthermore, a brief exposure to high levels of contamination increases the mortality of patients with a heart condition [1].

The PM is a mixture of solids and liquid droplets floating in the air. Such mixture is considered the severest air pollution problem due to its serious effects on human health [4]. Several studies have associated high concentrations of airborne PM with increased mortality and morbidity [5,6].

The metal content of PM contributes to its toxicity, by increasing the possibility of cardiopulmonary injuries [7]. It has been reported that the metals contained in air generate cardiovascular diseases, damage in brain function, lungs, liver and other organs [8]. In many countries, the highest metal content in the PM is mainly composed by lead (Pb) [9]. Thus, the PM size and, in general, the physicochemical characterization is necessary to understand the toxicology of particles [10]. Despite the existence of studies on the Pb effects on the cardiovascular system, the pathophysiological mechanisms of the alterations in the cardiac electrical activity in humans due to Pb exposition remain largely unknown. Bearing these ideas in mind, the aim of this work is to study the effects of Pb on cardiac arrhythmias using two- (2D) and three-dimensional (3D) models of the human atria. The Pb importance lies in the fact that it is the most abundant metal found in the PM characterization. The results of this study can contribute to the knowledge about the cardiac physiopathological processes, triggered by atmospheric pollutants, that yield the generation of arrhythmias.

## Materials and methods

This study includes two stages: a PM_10_ characterization step and computational simulations. For the first stage, PM_10_ samples were collected and a physicochemical characterization was performed. The computational simulations are designed for testing the electrophysiological effects of the most prevalent metal content from the collected PM_10_. For this purpose, a mathematical model of the pollutant is formulated, and it is coupled to human atrial virtual models, in which the electrophysiological features are measured. The following sections provide detailed information about the methodological procedures.

### Sample collection and extraction

The PM_10_ was collected by a certified monitoring station located at the University of Medellin in Medellín, Colombia. The sampling period was four days during the month of May 2018 and a PM_10_ Size Selective Sampling Inlet provided by Staplex was used. According to the EPA standard [11], the volumetric flow was adjusted between 1.02 m^3^/min and 1.24 m^3^/min. After the collecting period (24 hours), the filters were desiccated during 24 hours in a desiccator containing silica gel. The PM_10_ collected by the quartz microfiber filters was extracted by using Soxhlet extraction during 24 hours [12]. After that, the extract was concentrated using a rotary evaporator and dried at 80°C in an oven overnight.

### Physicochemical characterization

The morphology of PM was determined by a scanning electron microscope JEOL JSM-7100F (FE-SEM) with a voltage of 15 kV and a working distance of 10 mm. The PM chemical composition was obtained by Energy Dispersive Spectrometry (EDS) using a X-MAXN, OXFORD coupled to a scanning electron microscope with a voltage of 20 kV. Particles diameter was measured by the Image J free software. For this purpose, more than 580 particles were measured in 100 micrographs approximately. Heavy metals were determined and quantified using a Thermo Scientific^™^ iCAP^™^ 7000 Series ICP-OES (Inductivity Coupled Plasma Optical Emission Spectrometry) by the Standard Method 3120 A,B ed 23. Volatile fraction and elemental carbon in the PM were characterized by thermogravimetric analysis (TGA) using a TA Instruments SDT-Q600 with a ramp of 3°C per minute up to 450°C in a nitrogen atmosphere. Subsequently, it was changed to air atmosphere and a ramp of 5°C per minute up to 500°C [13].

### Human atrial cell model

The Courtemanche–Ramirez–Nattel–Kneller membrane formalism was implemented to simulate the electrical activity of human atrial cell [14,15]. The transmembrane voltage (*V*_*m*_) is given by the following equation:

$$C_{m}\frac{dV_{m}}{dt}+I_{ion}+I_{st}=0,$$ (1)

where *C*_*m*_ is the membrane capacitance (100 pF), *I*_*ion*_ is the total membrane current, and *I*_*st*_ is the external stimulus current. The model is parametrized for reproducing normal electrophysiological conditions.

### Model of the Pb effect

It has been reported that Pb^2+^ affects the cardiac electrical activity by blocking the L-type calcium channels (I_CaL_) [16]. The Hill’s equation has been used to fit the concentration-response relationships for I_CaL_ inhibition due to Pb^2+^. A basic formulation of the Pb effect on I_CaL_ was developed using the steady state fraction of blockade (*b*_*Pb*_):

$$b_{Pb}=\frac{1}{{\left[1+\frac{IC_{50}}{D_{Pb}}\right]}^{h}},$$ (2)

where *IC*_*50*_ is the half maximal inhibitory concentration for the current blockade by Pb^2+^ and *D*_*Pb*_ is the Pb^2+^ concentration. The Pb^2+^ was implemented using test concentrations values from 0 to 300 nM in incremental steps of 25 nM. The Hill coefficient (*h*) is set to *h =* 1, which indicates completely independent binding. A concentration of 152 nM was used for the *IC*_*50*_ to block I_CaL_, according to experimental studies [16]. The equations to calculate I_CaL_ are given by:

$$I_{CaL}=\left(1-b_{Pb}\right)g_{CaL}dff_{Ca}\left(V_{m}-E_{Ca}\right),$$ (3)

where *g*_*CaL*_ is the maximum conductance of I_CaL_, *d* is the activation gate, *f* is the voltage-dependent inactivation gate, *f*_*Ca*_ is the calcium-dependent inactivation gate and *E*_*Ca*_ is the equilibrium potential for calcium. Eq (3) was included in the atrial cell model to simulate the human atrial action potential under the presence of Pb^2+^.

### Atrial virtual models

A 2D model of human atrial tissue was designed as a 6 cm x 6 cm matrix, composed by 192 x 192 square elements. The domain was discretized with a spatial resolution of 312.5 μm. The tissue was considered isotropic. A conductivity of 0.4 S/cm was assigned in order to obtain a realistic conduction velocity (~ 60 cm/s).

**Fig 1:**
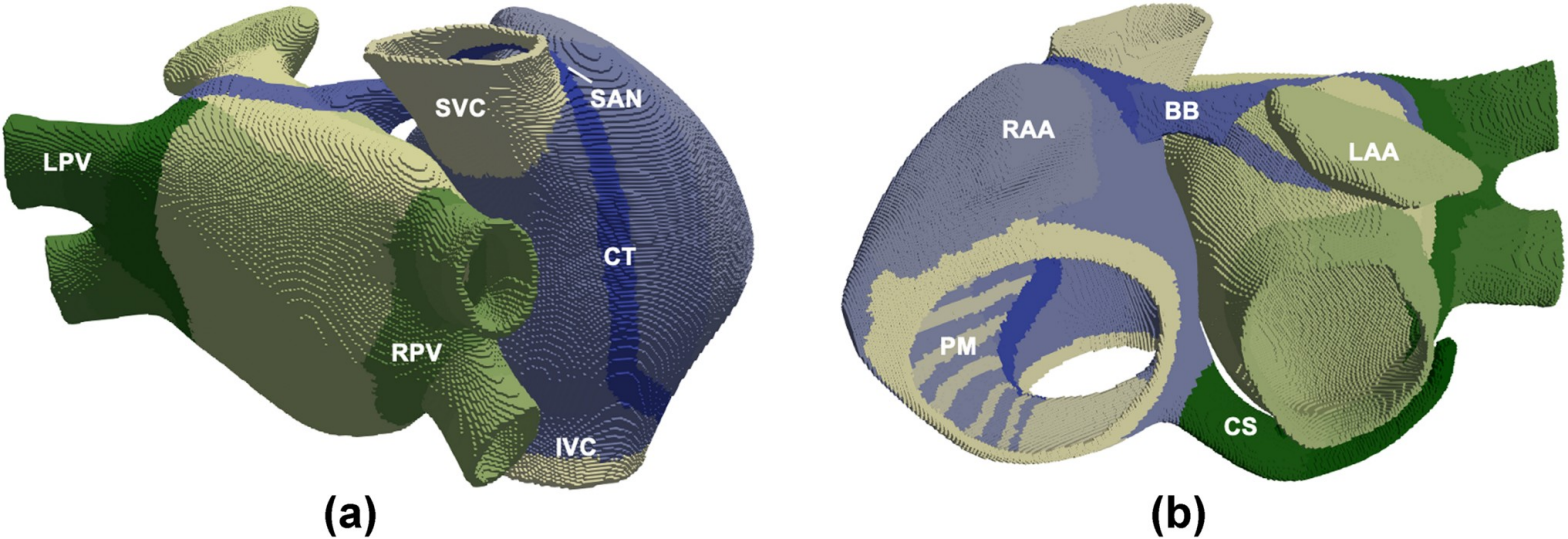
The 3D model of human atria. (**a**) Frontal and (**b)** dorsal views. Colored areas show regions with different fiber orientation, conductivity and/or electrophysiological heterogeneity. LAA and RAA: Left and right appendages, PM: Pectinate muscles, BB: Bachmann’s bundle, CS: Coronary sinus, LPV and RPV: Left and right pulmonary veins, SCV and ICV: Superior and inferior caval veins, CT: Crista terminalis, and SAN: Sinoatrial node.

A 3D model of human atria developed in previous work was also used [17]. The model includes the main anatomical structures (Fig 1), realistic fiber orientation, electrophysiological heterogeneity and anisotropy. This model is composed by 515,010 hexahedral elements with a uniform spatial resolution of 300 μm.

### Electrical propagation

The propagation in cardiac tissue is described by the monodomain equation of electrical propagation as follows:

$$\frac{1}{S_{v}}\nabla \cdot \left(D\nabla V_{m}\right)=C_{m}\frac{\partial V_{m}}{\partial t}+I_{ion}+I_{st},$$ (4)

where *S*_*v*_ is the surface/volume ratio and *D* is the conductivity tensor. The equation was numerically solved using the finite element method in the EMOS software [18].

### Stimulation protocols

After reaching the steady state of the atrial cell model, a train of 10 stimuli was applied at a basic cycle length of 1000 ms. The APD at 90% of the repolarization (APD_90_) and I_CaL_ were measured on the 10^th^ beat. The S1-S2 cross-field protocol was applied in the 2D model (rectangular pulses of 2 ms in duration and 6 mA in amplitude). The S1 is a stimulus applied at the left boundary of the tissue, aiming to generate a planar propagating wave. The S2 extrastimuli were applied at different times during the repolarization phase of S1. This protocol generates a gradient of excitation-refractoriness with the purpose of initiating reentrant activity. The time between S1 and S2 is called coupling interval and the vulnerable window is estimated as the period of time in which it is possible to generate a stable reentry sustained for at least 2 seconds.

The S1-S2 standard protocol was applied in the 3D model, where S1 corresponds to a simulated sinus stimulation in the SAN, and S2 is an ectopic focus applied at different coupling intervals in the repolarization phase of S1 and it is located at the cavotricuspid isthmus near to the coronary sinus. After applying the ectopic focus, 5 s of simulation are performed.

### Electrograms and dominant frequency

Pseudo-unipolar electrogram (EGMs) signals were recorded by virtual electrodes at 0.2 mm from the model surface. An EGM is calculated as the extracellular potential *ϕ*_*e*_ by applying the large volume conductor approximation:

$$\emptyset_{\text{e}}\left(\vec{r}\right)=-K\int \int \int \vec{\nabla^{\prime }}\;V_{m}\left(\vec{r}\prime \right)\cdot \vec{\nabla^{\prime }}\left[\frac{1}{\left|\vec{r}\prime -\vec{r}\right|}\right]dv,$$ (5)

where *K* (−0.0398) is a constant representing the ratio of intracellular and extracellular conductivities, ${\overset{⃑}{\nabla }}^{\prime }V_{\text{m}}$ is the spatial gradient of transmembrane potential, ${\overset{⃑}{r}}^{\prime }-\overset{⃑}{r}$ is the distance from the source point (*x’*, *y’*, *z’*) to the measuring point (*x*, *y*, *z*) and *dv* is the volume differential. The EGMs at different points were visually inspected in order to analyze their morphologies. Spectral analysis of the EGMs was performed by applying a 40–250 Hz band-pass filter, rectification and low-pass filter at 20 Hz. The discrete Fourier transform was obtained and the dominant frequency (DF) was identified as the frequency corresponding to the highest peak of the power spectrum.

## Results

### Physicochemical characterization

**Fig 2:**
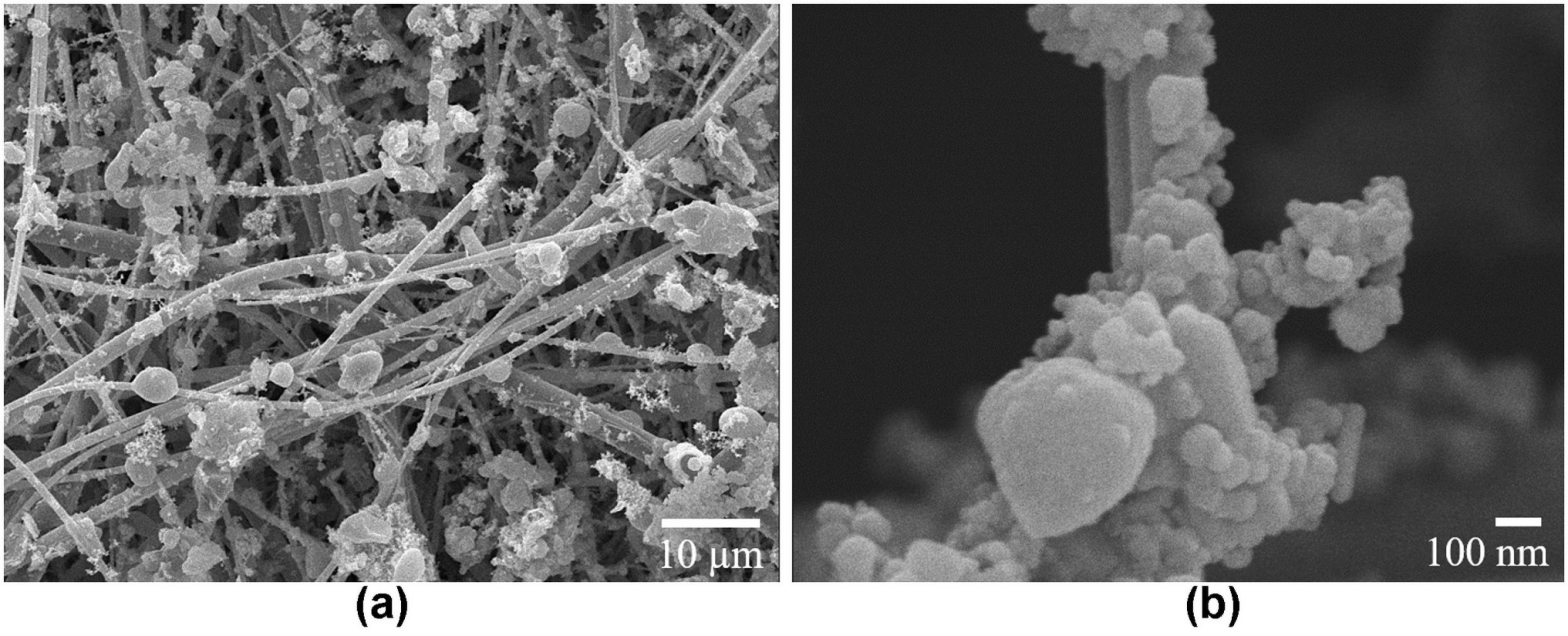
SEM of PM. Scanning electron micrographs of quartz microfiber filters before particulate matter extraction at (**a**) low and (**b**) high magnifications.

The PM morphology and size were studied by SEM. Fig 2a and 2b show the SEM micrographs of PM in one of the filters before the extraction process at different magnifications. The micrographs show different particulate matter attached to the filter fibers, mainly with a semispherical morphology. Additionally, the size of the particles in the SEM images was measured by the software Image J. The particles exhibit an D90 size of 4.25 μm with a minimum and maximum of 0.042 μm and 9.7 μm, respectively. The PM particles with aerodynamic diameter of 10 μm or less (PM_10_), 2.5 μm or less (PM_2.5_) and 0.1 μm or less (PM_0.1_), are classified as coarse, fine and ultrafine, respectively [10]. Our data suggests that the PM sizes can be classified as coarse (PM_10_). It is important to highlight that many of the observed particles are within the size range of the most harmful PM whose size is under 2.5 μm.

**Fig 3:**
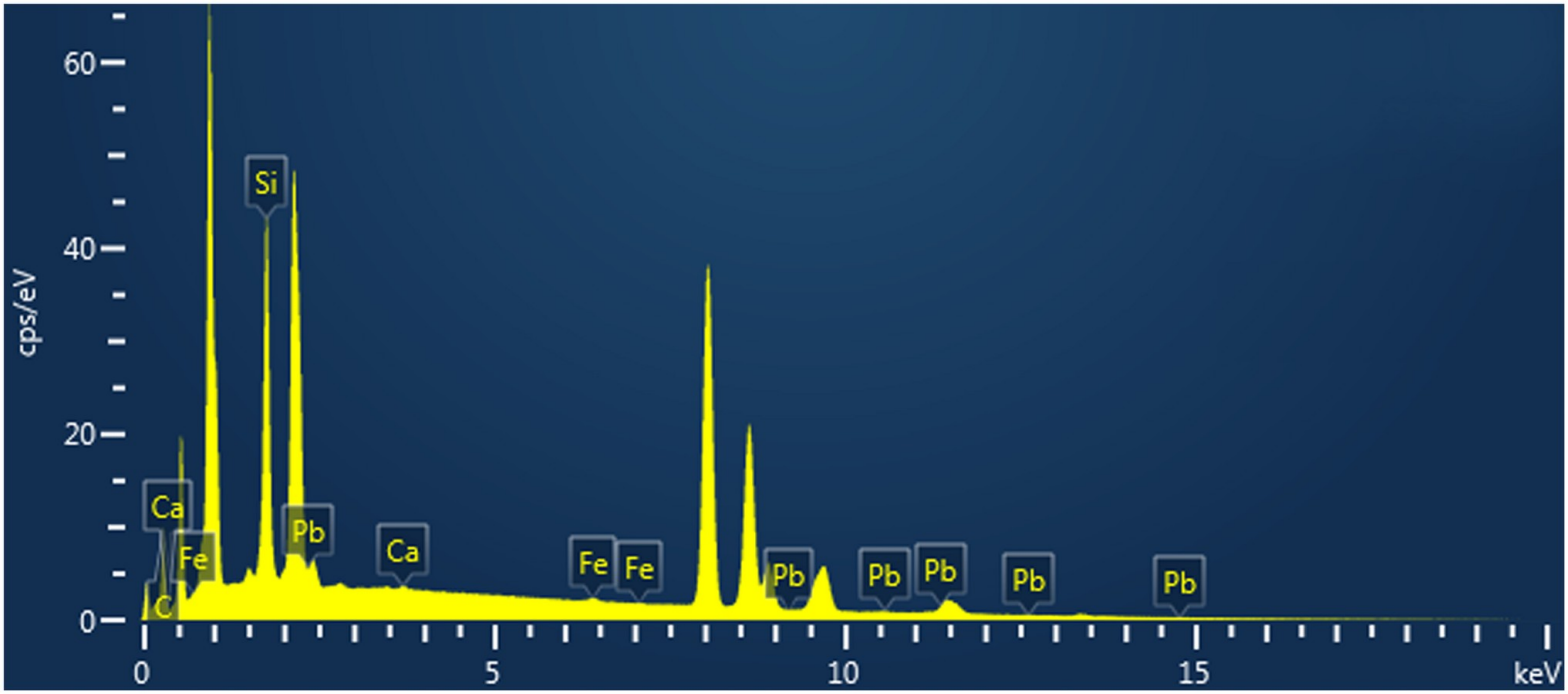
EDS of PM. Energy dispersive spectrum of particulate matter.

The particulate matter chemical composition was obtained by EDS analysis after the PM extraction process (see supplementary S1 Data). Fig 3 shows an EDS spectrum of the PM where the presence of elements such as carbon (C), silicon (Si), lead (Pb), calcium (Ca) and iron (Fe) can be observed.

Table 1 shows the weight percentage (wt %) of the elements found in the sample by EDS analysis. Carbon, silicon and lead are the most abundant elements present in all samples. Carbon is associated with vehicular, industrial, and domestic emissions [19], silicon is associated with detached filter fibers after the washing process and is was not possible to separate them from the PM; and lead is among the most harmful heavy metal caused from vehicles and industry sources, consequently it needs special attention [20]. Moreover, the presence of calcium could be related with the existence of a quarry located 145 m form the sampling point [21]. Iron was also identified in the analysis, as it is abundant in industrial processes of the industrialized cities [22]. In addition, the analysis performed by Inductivity Coupled Plasma Optical Emission Spectrometry (ICP-OES), confirmed the presence of lead, with a concentration of 6940 nM.

**Table 1:** EDS compositional results.

Element	wt%	wt% Sigma
**C**	54.82	0.65
**Si**	34.71	0.48
**Pb**	6.69	0.58
**Ca**	1.95	0.11
**Fe**	1.83	0.21

**Fig 4:**
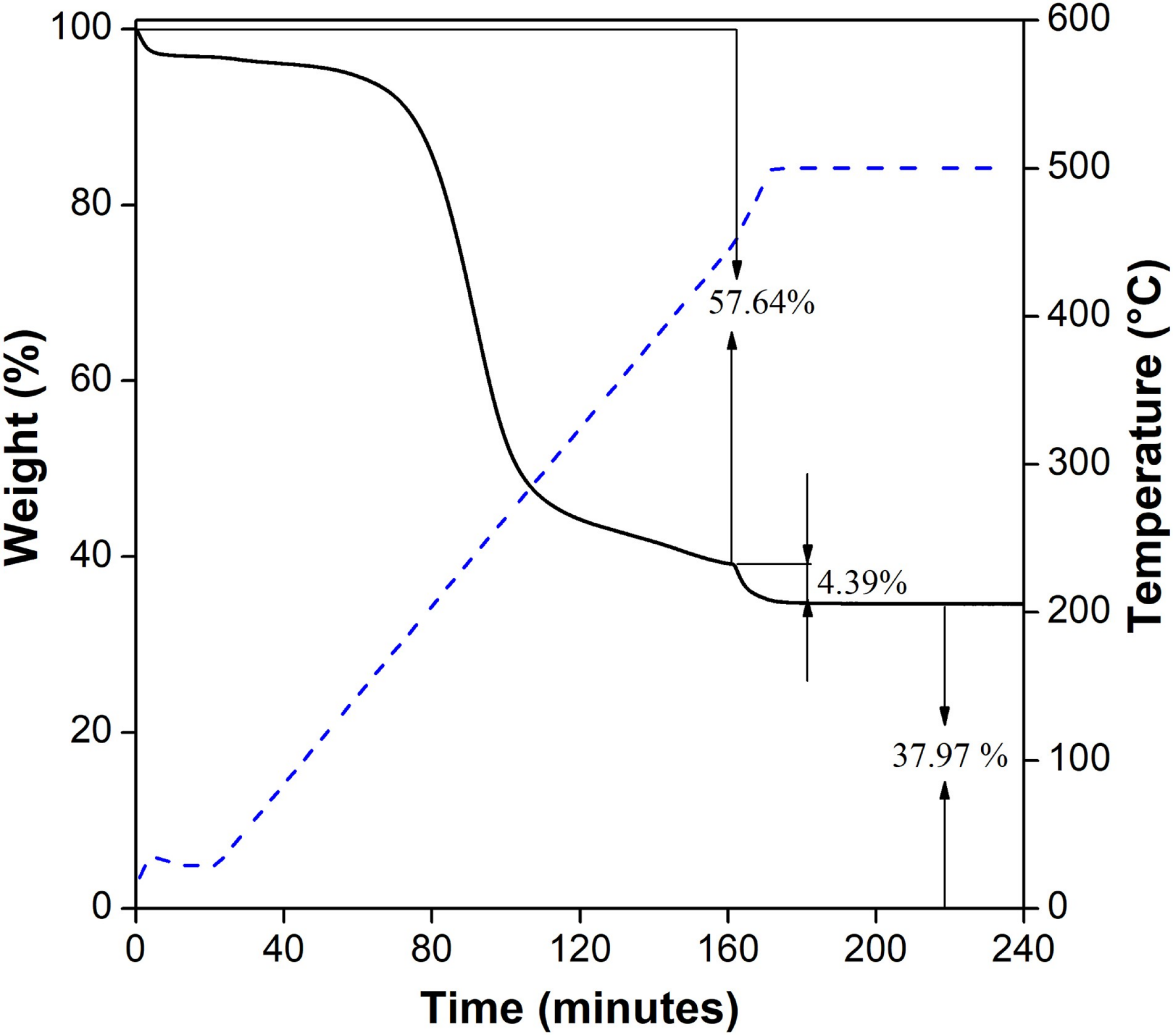
TGA of PM. Thermogravimetric analysis of particulate matter.

After the PM extraction, humidity, volatile material and elemental carbon were determinated by TGA. Fig 4 shows the obtained data, in which two thermal events are identified. First, an initial weight loss (57.64%) between 100°C and 440°C, that corresponds to water adsorbed by the material, and it could also be related to the loss of volatile material such as the degradation of fossil fuels and lubricant oil residues from vehicles [23]. A second thermal event, around 462°C, exhibits a weight loss of 4.39%. Such weight loss is related with elemental carbon emitted during the incomplete combustion of fossil fuels and biomass burning [24]. The remaining weight after the analysis (37.97%) is mainly related with silicon from the filter and other components as Pb.

### Pb effect on atrial activity

**Fig 5:**
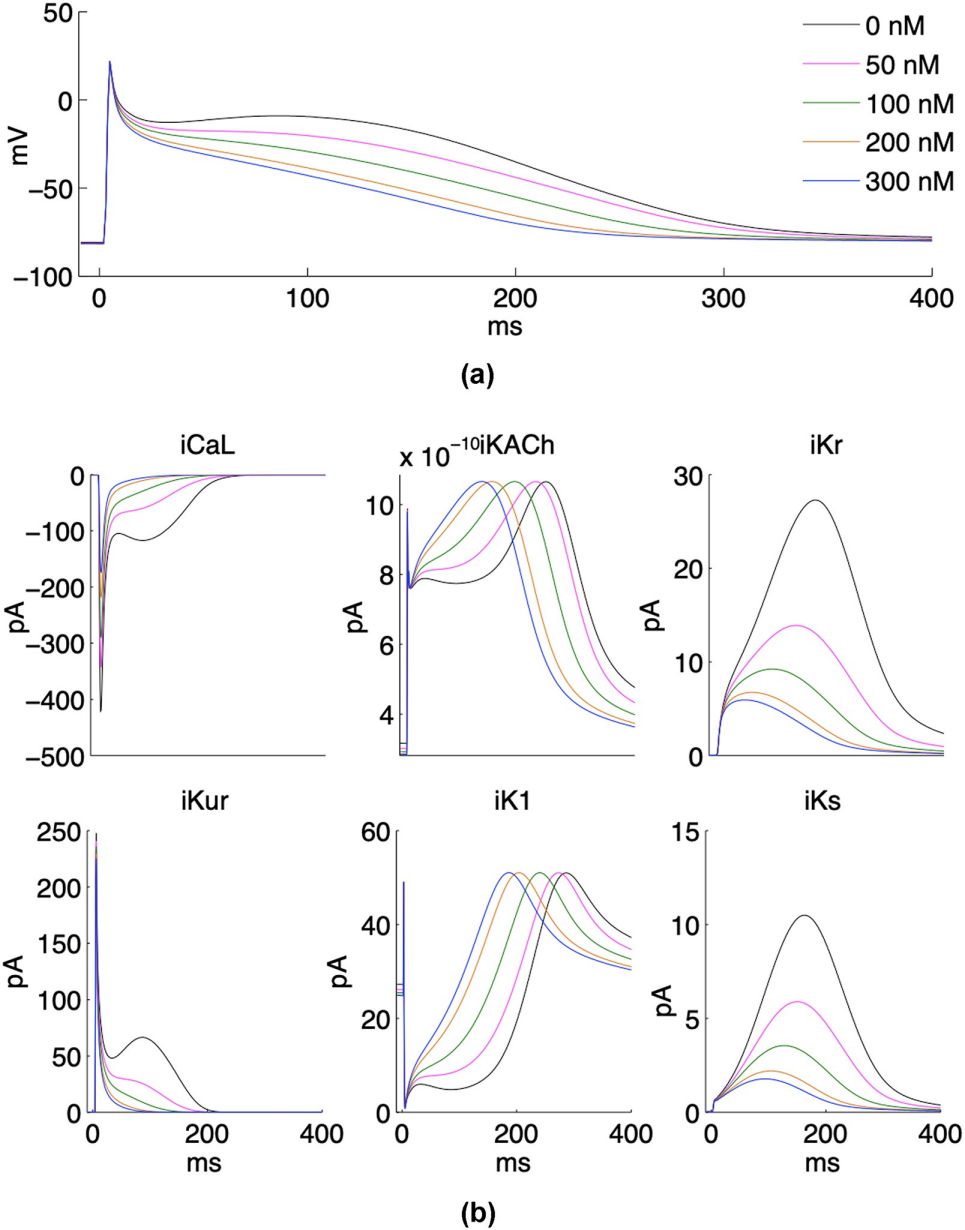
Atrial action potential and the main ionic currents at different Pb^2+^ concentrations. As the Pb^2+^ concentration increases, (**a**) the action potential loses the dome of the plateau phase and the APD is shortened; and (**b**) downregulation or/and curves displacements of the main ionic currents are observed.

The results of the PM characterization showed high concentrations of C, Si, and Pb. Since carbonaceous materials have been widely studied and Si is mostly related to the filter material, Pb was selected to study its proarrhythmic effect on atrial activity by computer simulations. Fig 5 shows the effects of different Pb^2+^ concentrations on the atrial action potential (Fig 5a) and the main ionic currents (Fig 5b), the curves correspond to the results obtained for concentrations in incremental steps of 100 nM for a better visualization of the Pb^2+^ effect. Under the absence of Pb^2+^ (0 nM), the action potential presents a pronounced plateau phase (known as dome shape) with an APD_90_ of 314 ms. The maximum peak of the ionic currents is -422 pA for I_CaL_, 27 pA for the rapidly activating potassium current (I_Kr_), 247 pA for the ultrarapid delayed rectifier potassium current (I_Kur_) and -174 pA for the slowly activating potassium current (I_Ks_). The acetylcholine-dependent potassium current (I_KACh_) and the inwardly rectifying potassium current (I_K1_) have maximum peaks during the plateau phase of the action potential, at 249 ms and 296 ms respectively (taking 0 ms as the time where the 10^th^ beat begins).

As the Pb^2+^ concentration increases, a downregulation of I_CaL_ is observed. This effect on calcium current leads to a downregulation of I_Kr_, I_Kur_ and I_Ks_ currents, and a shortening of the I_KACh_ and I_K1_ transient duration, which causes an APD shortening and the loss of the dome of the plateau phase. As the Pb^2+^ concentration increases, a significant shortening in APD was observed (see Table 2). When the highest Pb^2+^ concentration was applied (300 nM), the I_CaL_, I_Kr_, I_Kur_ and I_Ks_ maximum peaks showed values of -174 pA, 6 pA, 225 pA and 2 pA, indicating a decrease of 59%, 78%, 9% and 80%, respectively; the I_Kr_ and I_K1_ transient duration are reduced to 109 ms and 99 ms (maximum peaks at 140 ms and 197 ms), respectively; the I_Kr_ and I_Ks_ peak also presented short transients of 120 ms and 69 ms; and the APD_90_ reached a value of 216 ms, which indicates a decrease of 31%.

**Table 2:** Main quantitative results from 2D and 3D simulations.

[Pb^2+^] (nM)	↓ APD_90_	↓ CV	2D model			3D model		
			CI (ms)	VW (ms)	DF (Hz)	CI (ms)	VW (ms)	DF (Hz)
175	21%	3%						
200	26%	5%	209–211	3	7.3			
225	27%	5%	206–209	4	7.3			
250	28%	6%	202–209	8	7.5	212–216	5	6.0
275	29%	6%	198–208	11	7.5	213–219	7	6.0
300	31%	6%	192–206	15	7.5	213–220	8	6.0

APD_90_ and conduction velocity (CV) reductions, coupling intervals (CI), vulnerable windows (VW) and dominant frequencies (DF) obtained in the 2D and 3D models for Pb^2+^ concentrations [Pb^2+^] between 175 nM and 300 nM. Reduction: **↓**.

**Fig 6:**
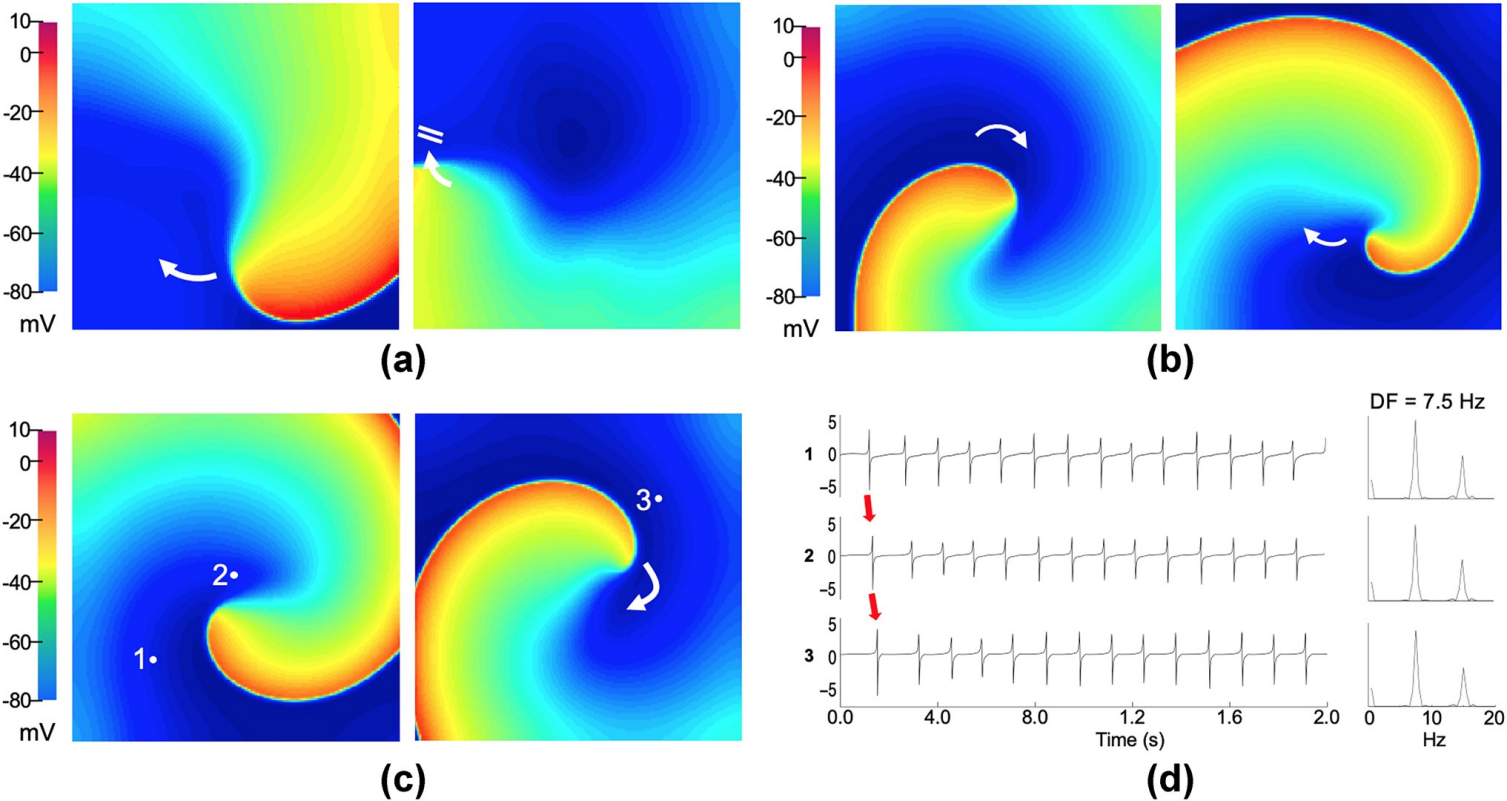
Computational simulations in the 2D atrial model. Snapshots of membrane voltage during (**a**) wave blockade at Pb^2+^ concentration of 175 nM, (**b)—(c**) stable rotor-type reentries (curved arrows indicate rotation direction) obtained at a coupling interval of half of the vulnerable window by applying Pb^2+^ concentrations of 200 nM and 300 nM, respectively. (**d**) EGMs and their spectra at three selected points (1 to 3 from Fig c), where the activation sequence of the reentry on its first turn is indicated by the red arrows. The DF value of 7.5 Hz is displayed.

In the 2D model of human atrial tissue, the conduction velocity was calculated during S1 stimulus application. The conduction velocity obtained under absence of Pb^2+^ was 64.5 cm/s, as the Pb^2+^ concentration was increased, a slight conduction velocity reduction was observed, reaching a value of 60.6 cm/s (6% decrement) at the highest Pb^2+^ concentration (300 nM). After applying the S1-S2 cross-field protocol to the 2D model, it was not possible to generate a reentrant activity using the Pb^2+^ test concentrations values lesser than 200 nM (i.e., 175 nM or lowers). The wavefront generated by S2 rotates on itself, but it collides with its own refractory tail (unexcitable tissue) and it extinguishes because the refractory period is greater than the rotational trajectory (Fig 6a). On the other hand, when we applied Pb^2+^ concentrations greater or equal than 200 nM, the wavefront path (having a shorter refractory period) encounters excitable tissue and its rotation continues on itself, generating a stable rotor-type reentry in the tissue. For the Pb^2+^ concentration of 200 nM, rotors were generated within S1-S2 coupling intervals from 209 ms to 211 ms, obtaining a vulnerable window of 3 ms. Fig 6b shows snapshots of membrane voltage during a stable rotor obtained at a coupling interval of 210 ms (half of the vulnerable window). As the Pb^2+^ concentration increased, the vulnerable window to reentry increased (see Table 2). For the highest Pb^2+^ concentration (300 nM), rotors were generated within S1-S2 coupling intervals from 192 ms to 206 ms, obtaining a vulnerable window of 15 ms. Fig 6c shows a stable rotor obtained at a coupling interval of 199 ms (half of the vulnerable window). The tip of the reentrant wave rotates in a circular orbit in the center of the tissue. The arrow in the figure shows the clockwise direction of rotation. Fig 6d shows three EGMs and their spectra calculated at the points indicated in the Fig 6c. The red arrows indicate the activation sequence of the reentry wavefront during the first full rotation. The EGMs display single potentials, indicating a stable and regular activation of the atrial tissue. Such morphological regularity is reflected as a single narrow DF peak of 7.5 Hz throughout the atrial tissue, which is characteristic of tachycardias sustained by a focal activity or stable reentry.

**Fig 7:**
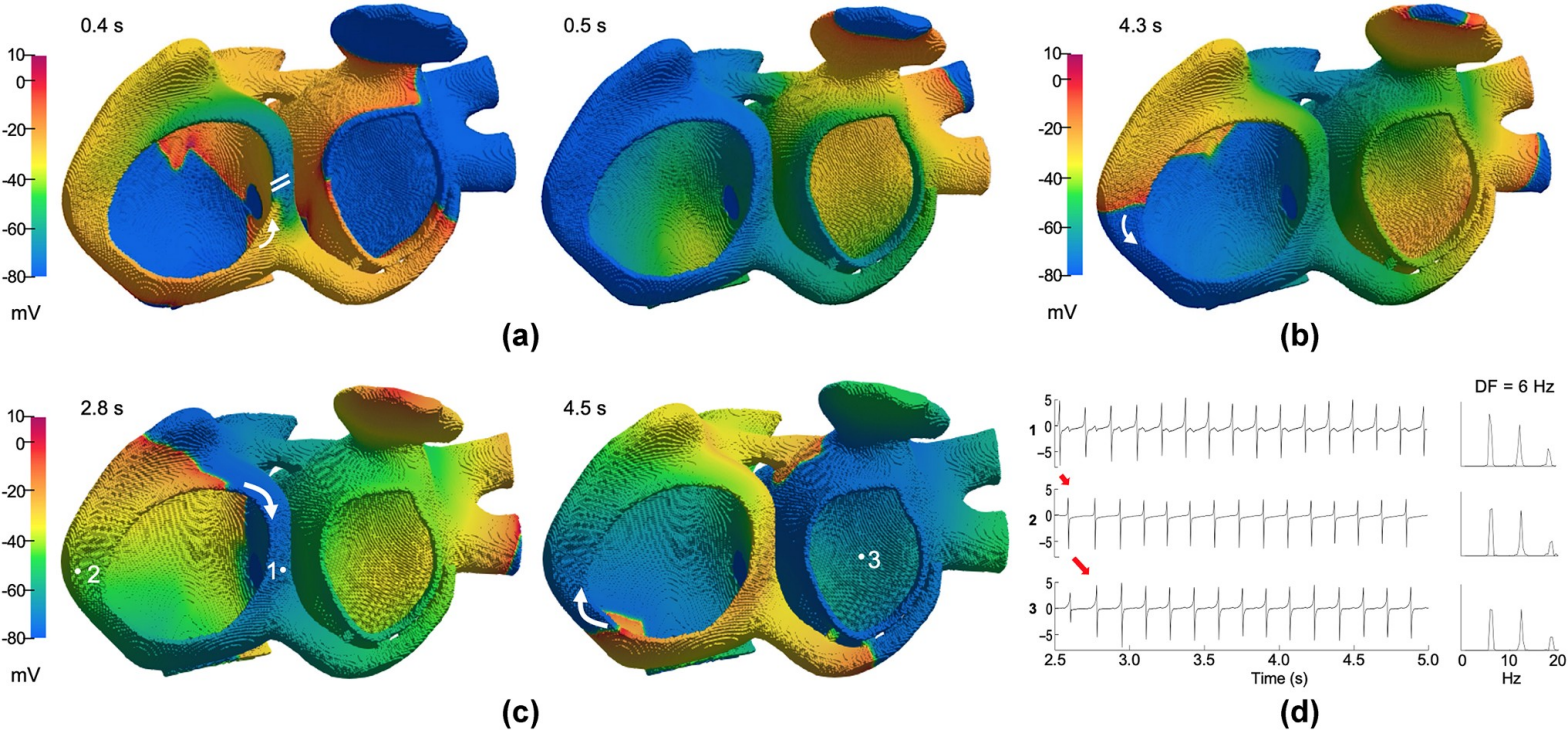
Computational simulations in the 3D human atrial model. Snapshots of membrane voltage showing the S2 wavefront initiation by a unidirectional block. Each panel depicts the evolution of the propagation for different Pb^2+^ concentrations. The wavefront propagates through the tricuspid ring and then, (**a**) blocks upon encountering refractory tissue for the Pb^2+^ concentration of 225 nM; (**b**) generates a typical atrial flutter (curved arrows indicate rotation direction) for the Pb^2+^ concentration of 250 nM; (**c**) generates a reverse typical atrial flutter for the highest concentration of Pb^2+^ (300 nM). (**d**) EGMs and their spectra at three selected points (1 to 3 from Fig c). The activation sequence of the flutter is indicated by the red arrows. The DF value of 6 Hz is displayed.

By applying the S1-S2 protocol to the 3D model of human atria, for Pb^2+^ concentrations from 0 to 225 nM, atrial arrhythmias cannot be generated. The S2 wavefront initiated by a unidirectional block at different coupling intervals, propagates through the tricuspid ring. However, as the APD was not short enough, the wavefront reached refractory tissue and became extinct (Fig 7a). On the contrary, for the higher Pb^2+^ concentrations (250 nM, 275 nM and 300 nM), typical atrial flutter episodes sustained by a macro-reentry through the tricuspid ring in the right atrium were observed. As the Pb^2+^ concentration increased, the vulnerable window to reentry increased (see Table 2). Fig 7b and supplementary S1 Video show a typical atrial flutter by applying Pb^2+^ concentration of 250 nM and an S2 at a coupling interval of 214 ms (half of the vulnerable window), where the macro-reentry presents a constant cycle in the counterclockwise around the tricuspid ring. For the highest Pb^2+^ concentration (300 nM), stable macro-reentries were generated within S1-S2 coupling intervals from 213 ms to 220 ms, obtaining a vulnerable window of 8 ms. Fig 7c and supplementary S2 Video show a reverse typical atrial flutter generated at a coupling interval of 216 ms (half of the vulnerable window), where the wavefront propagated in the counterclockwise direction around the tricuspid ring at the beginning of the simulation, but then, a retrograde stimulus on the right atrium floor caused a change in the rotation direction. Such events lead to the generation of a macro-reentry rotating in the clockwise direction around the tricuspid ring (see the white arrows in Fig 7c), that perdured during the rest of the simulation. The electrical impulse travels up the right atrium from the coronary sinus and the tricuspid valve, activating the right and left atrium at high frequency. The direction of the reentrant circuit can also be observed when comparing the EGMs of the sites 1, 2 and 3 in Fig 7d (see red arrows). The macrorentrant wavefront depolarized the rest of the atria, including the posterior wall of the left atrium (site 3 in Fig 7c) with a one-to-one activation pattern. The cycle length of this arrhythmic pattern was constant (< 200 ms) in the whole atria, which is characteristic of atrial flutter. The EGMs recorded at different points of the atria only display single potentials, showing a stable and regular atrial activation, which is also a typic feature of flutter. The EGM regularity is reflected as a single narrow DF peak of 6.0 Hz within the whole spectra.

Table 2 summarizes the main in silico results including, the reductions in APD_90_ and conduction velocity in percentage values, and the coupling intervals, vulnerable windows and dominant frequencies obtained from the 2D and 3D simulations. These results correspond to Pb^2+^ concentrations between 175 nM and 300 nM. A total of 41 rotors were obtained in the 2D model for Pb^2+^ concentrations between 200 nM and 300 nM, and 20 flutter episodes in the 3D model for concentrations between 250 nM and 300 nM. In all simulations, an increase in vulnerability to reentry was observed as the Pb^2+^ concentration increases.

## Discussion

The physicochemical characterization of the PM collected showed semispherical particles with sizes between 0.042 μm and 9.7 μm, where the main metallic element found was Pb, therefore, it was selected to study its effect on atrial activity. Our simulations in 2D and 3D models of human atria shown a concentration-dependent proarrhythmic effect of Pb, expressed through the APD shortening and the generation of reentries and atrial flutter at high Pb concentrations.

Epidemiologic studies have shown a correlation between the increased mortality and the increment of PM in the air, where the mortality rising numbers are mainly related to cardiovascular events [25–27]. It has been shown that prolonged exposures to pollutants reduce people life expectancy by several years, and hospital admissions due to cardiovascular diseases increase with high pollutants concentrations in the environment [1]. Moreover, a higher probability of cardiac arrhythmias appearance and a mortality risk of 76% related with cardiovascular disease after exposure to atmospheric pollutants have been reported [28,29]. Deaths are commonly related to ischemia, arrhythmias and heart failure [30]. Short-term exposure to PM may be relevant to events causing myocardial infarction [26] and heart rate alteration [31]. On the other hand, long-term exposure to PM air pollution was associated with an increased risk of total mortality and cardiovascular disease [32]. Our results support the idea that high concentrations of toxic material in the atmosphere could be the cause of the increase in cardiovascular mortality.

Several studies have reported alterations in cardiac function in animals exposed to PM, providing information of the toxicity mechanisms of PM [33]. An experimental study with 32 rats, showed that exposure to PM combined with residual oil fly ash causes premature ventricular and atrial arrhythmias [34]. An investigation with 12 rats exposed to PM through endotracheal intubation, evinced a relationship between cardiovascular disease and PM exposure, in which, premature ventricular contractions, ventricular tachycardia and increased PR and QT interval were observed [35]. Another study in rats exposed to PM described an increment of heart vulnerability to cardiac arrhythmias, by means of electrograms analysis [36]. An experimental study with 152 dogs from different cities of Mexico was conducted: a group of 109 belonged to highly polluted cities in Mexico and a group of 43 to less polluted cities. The results showed that the group residing in cities with lower levels of pollutants have little or none cardiac abnormalities, while the remaining group showed myocardial alterations including apoptotic myocytes and severe vascular changes [37]. A follow-up clinical study was conducted in 176 patients with known cardiac diseases during an average of 1.9 years. The study concluded that PM is an acute trigger of atrial fibrillation [28].

The high concentrations of Pb obtained from the physicochemical characterization of PM_10_ is in agreement with studies showing high atmospheric Pb concentration with an PM_10_ average of 540 ng/m^3^ [38], that exceeds the air quality standard of Pb (200 ng/m^3^) recommended by the World Health Organization. Factories and vehicles arise as big contributors of PM within the city, a previous study reveals that during 2015, factories emitted 56 kg of Pb and 144.9 tons of PM_10_ [39]. In addition, an important part of the vehicles in the city exhibit a significant engine technological backwardness, which favors the emission of PM [40]. The Pb has been shown as the most dangerous heavy metal, able to cause harmful effects in human beings [41]. The exposure to Pb drive severe physiological effects even at low exposition levels [42] and it is associated with an increment of blood pressure, myocardial infarction and stroke mortality [43]. Our in-silico results showed that Pb^2+^ blocks the I_CaL_ current in a larger fraction as the Pb^2+^ concentration increases, extending its effect in the time through APD shortening, which is in agreement with experimental studies. The calcium channel blockade by Pb has been reported in other cell types [44,45]. In cardiac tissue, an study in ventricle myocytes of rats showed that Pb^2+^ blocks the L-type calcium channels [16]. However, the authors claim that the underlying mechanism are not clearly understood. Ferreira de Mattos et al. [46], in isolated cardiomyocytes and isolated guinea pig hearts, reported that Pb was cardiotoxic and reduced cardiac contractility, making the heart prone to arrhythmias. This was, in part, due to the effects of extracellular lead in blocking calcium currents through Cav1.2 channels. Another study with the rat ventricular myocardium, suggests that acute administration of Pb^2+^ reduces the myocardial contractility. During this affectation, the Pb^2+^ reduces the sarcolemic calcium influx and myosin ATPase activity, which was experimentally demonstrated [47].

In this study, a total of 41 stable rotors in the 2D model for Pb^2+^ concentrations between 200 and 300 nM, and 20 flutter episodes in the 3D model for concentrations between 250 and 300 nM, where found. In both cases, an increase in vulnerability to reentry was observed as the Pb^2+^ concentration increases. Rotors have been widely reported to be important mechanisms maintaining atrial arrhythmias [48,49]. Additionally, typical atrial flutter is an arrhythmia with a well-known reentrant mechanism and relatively frequent in clinical practice [50,51]. The difference observed between the Pb^2+^ concentration values that yield sustained reentries, and the differences in the vulnerable windows and dominant frequencies, could be explained given that the 2D model represents a simplified isotropic and electrophysiologically homogeneous domain, whereas, the 3D model is a highly detailed and realistic model with electrophysiological heterogeneity, with sectorized anisotropy and conductivity. Moreover, the stimulation protocols for reentries generation applied to both models are different due to their structural characteristics, which leads the initiation of different arrhythmic episodes. Therefore, the outcomes obtained in our 2D and 3D simulations related to the APD shortening, slight conduction velocity reduction, generation of arrhythmic mechanisms (rotors and flutters), and longer vulnerable windows, suggests a concentration-dependent proarrhythmic effect of Pb as a component present in atmospheric pollution.

Further research is required to fully unveil the cardiopathologic mechanisms triggered by PM pollutants, specifically on the Pb effect in the atrial tissue. In silico studies may contribute to a better understanding the mechanisms by which PM has unhealthy effects on cardiac tissue, promoting cardiac diseases such as arrhythmias.

### Limitations

One of the main limitations in the characterization of PM was to achieve detangling of the filter particles. This problem was solved using the Soxhlet method, in this method the filters are repeatedly washed with a hot solvent to completely remove the particles. Furthermore, the heterogeneity of the sample could be a problem, to avoid this error the analysis was carried out for four days.

Our in silico study has some methodological limitations: due to the lack of data from studies in atrial myocytes, our formulation uses values of IC_50_ from studies in ventricular myocytes. The evaluation of the Pb^2+^effect on the action potential and on the generation of reentries in the 2D and 3D models was performed by testing concentrations from 0 to 300 nM in incremental steps of 25 nM. Such value was considered since the changes in the action potential do not exceed 5% between concentrations, however, complementary studies using different test values and higher concentrations would be necessary. Additionally, the spiral wave generation was assessed under variations in the concentration of Pb^2+^ and the coupling interval. Future work could evaluate alterations in the initial conditions or parameters of the model and their effect on the probability of generating a spiral wave. Regarding the S1-S2 protocol, the configuration of the S2 stimulus adopted in this work is not the unique option. Different configurations for the S2 stimulus in the 2D and 3D models could trigger other types of reentries, such as figure-of-eight reentries in the 2D model or episodes of tachycardia or atrial fibrillation in the 3D model. Future studies could assess the resulting dynamics under distinct S2 extrastimuli setups.

## Conclusion

In this work, the PM_10_ collected was physicochemically characterized. The Pb was found as the most abundant metal element present in the collected samples. Moreover, the simulations in 2D and 3D models of human atria during normal electrophysiological conditions, showed that high concentrations of Pb leads to APD shortening, and reentrant mechanisms and atrial flutter. These features suggest that the Pb have a relevant proarrhythmic effect in the atria. Further computational studies may contribute to improve the understanding of the mechanisms by which air pollutants promoting cardiac arrhythmias.

## Supporting information

S1 Data EDS analysis of particulate matter. Spectrum and data set of the particulate material analyzed by EDS. All the analysis were performed at 20 keV and WD:10.
(PDF)

S1 Video Typical atrial flutter. Computational simulation in the 3D human atrial model showing a typical atrial flutter generated under a Pb^2+^ concentration of 250 nM.
(M4V)

S2 Video Reverse typical atrial flutter. Computational simulation in the 3D human atrial model showing a reverse typical atrial flutter generated under the highest concentration of Pb^2+^ (300 nM).
(M4V)
