# Supplementary material for: Atrial proarrhythmic effect of lead as one of the PM10 metal components of air pollution. An in-silico study
Source: PLoS One. 2021 Oct 12;16(10):e0258313. doi: 10.1371/journal.pone.0258313 (PMC8509962; doi:10.1371/journal.pone.0258313)
Supplement: S1 Data — Spectrum and data set of the particulate material analyzed by EDS. All the analysis were performed at 20 keV and WD:10. (PDF) [file pone.0258313.s001.pdf]

Electron Image 17

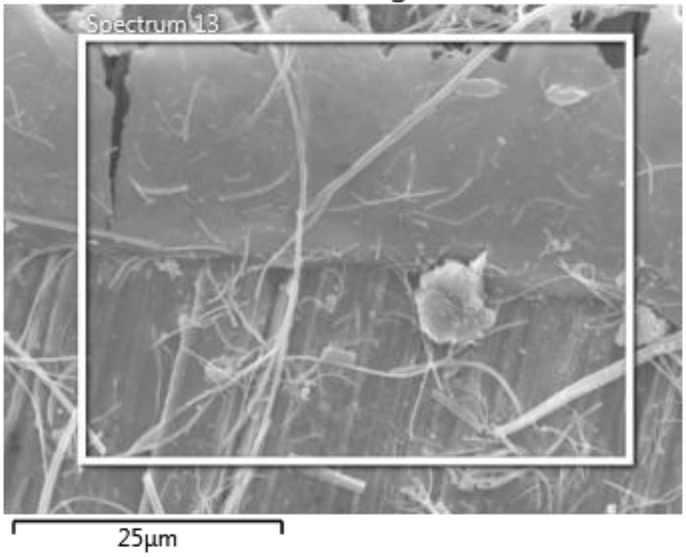

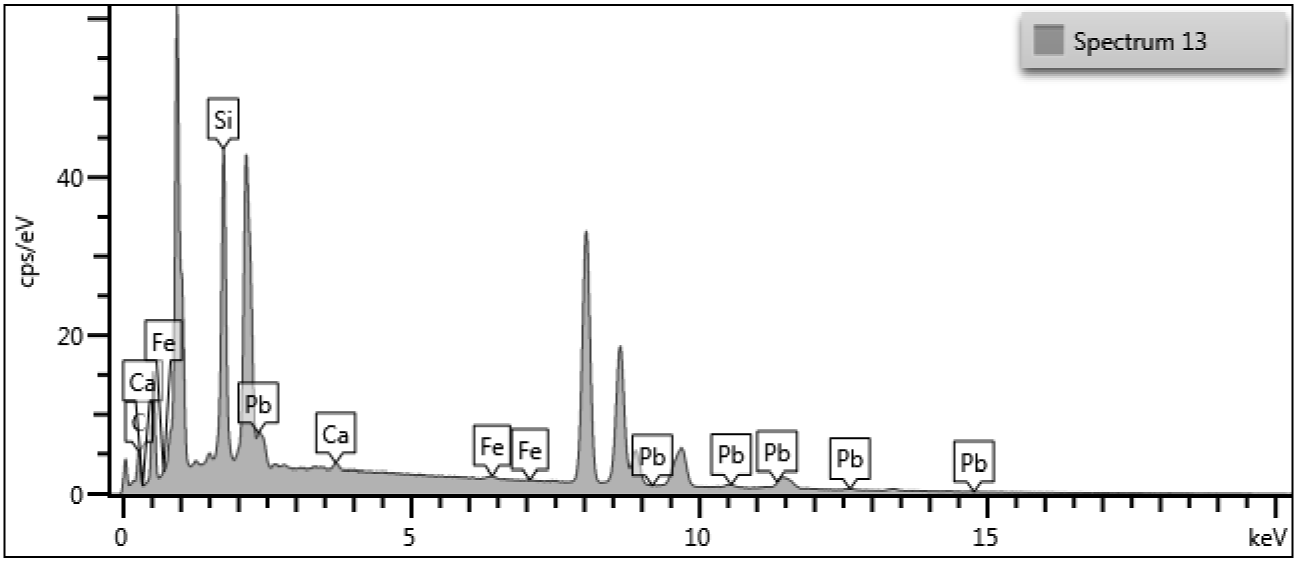

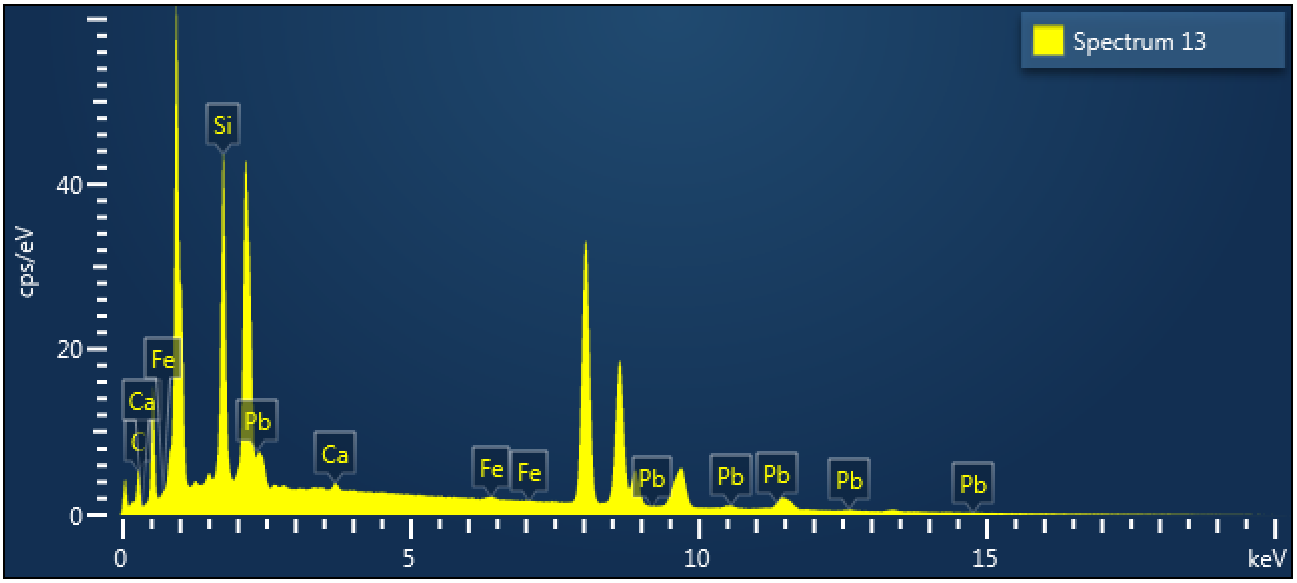

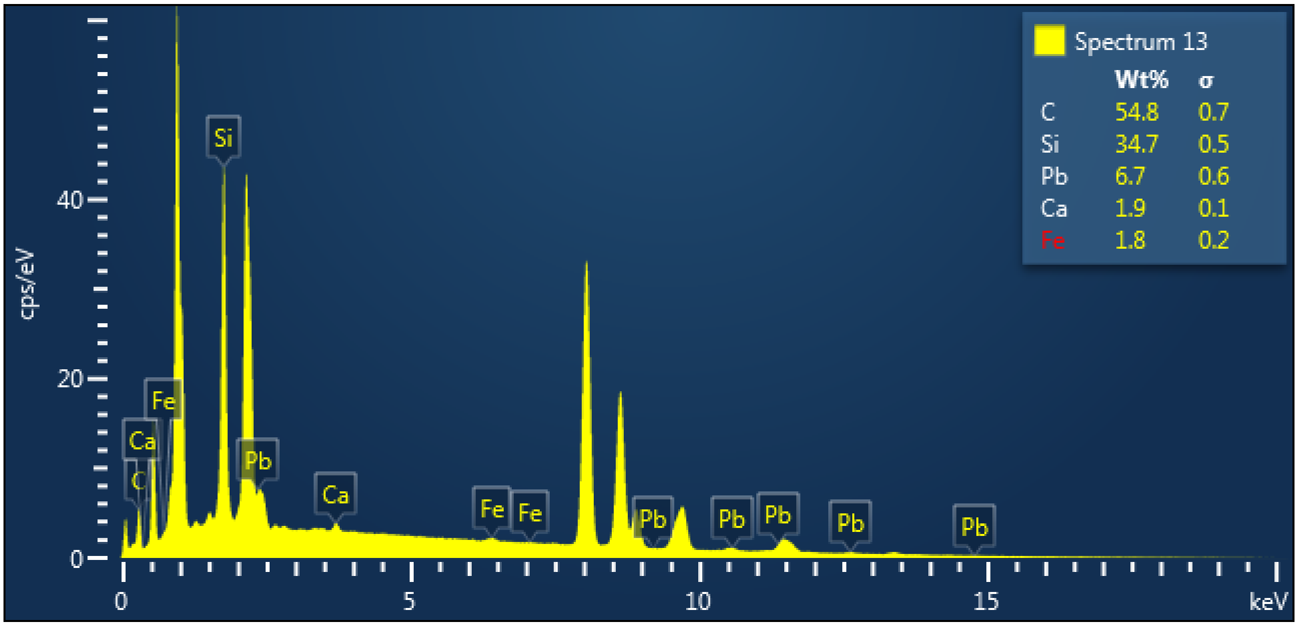

Element	Wt%	Wt% Sigma
C	54.82	0.65
Si	34.71	0.48
Ca	1.95	0.11
Fe	1.83	0.21
Pb	6.69	0.58
Total:	100.00	

Element	Line Type	Apparent Concentration	k Ratio	Wt%	Wt% Sigma	Standard Label	Factory Standard	Standard Calibration Date

C	K series	1.52	0.01519	54.82	0.65	C Vit	Yes	
Si	K series	7.88	0.06245	34.71	0.48	SiO_2	Yes	
Ca	K series	0.38	0.00335	1.95	0.11	Wollastonite	Yes	
Fe	K series	0.31	0.00313	1.83	0.21	Fe	Yes	
Pb	M series	1.05	0.00978	6.69	0.58	PbTe	Yes	
Total:				100.00				
